# The role of soil microbes in the global carbon cycle: tracking the below-ground microbial processing of plant-derived carbon for manipulating carbon dynamics in agricultural systems

**DOI:** 10.1002/jsfa.6577

**Published:** 2014-03-06

**Authors:** Christos Gougoulias, Joanna M Clark, Liz J Shaw

**Affiliations:** Soil Research Centre, Department of Geography and Environmental Science, School of Archaeology, Geography and Environmental Science, University of ReadingRG6 6DW, United Kingdom

**Keywords:** carbon cycling, rhizosphere carbon flow, decomposition, soil microbial respiration, climate change, methods, carbon tracking, agro-ecosystem management

## Abstract

It is well known that atmospheric concentrations of carbon dioxide (CO_2_) (and other greenhouse gases) have increased markedly as a result of human activity since the industrial revolution. It is perhaps less appreciated that natural and managed soils are an important source and sink for atmospheric CO_2_ and that, primarily as a result of the activities of soil microorganisms, there is a soil-derived respiratory flux of CO_2_ to the atmosphere that overshadows by tenfold the annual CO_2_ flux from fossil fuel emissions. Therefore small changes in the soil carbon cycle could have large impacts on atmospheric CO_2_ concentrations. Here we discuss the role of soil microbes in the global carbon cycle and review the main methods that have been used to identify the microorganisms responsible for the processing of plant photosynthetic carbon inputs to soil. We discuss whether application of these techniques can provide the information required to underpin the management of agro-ecosystems for carbon sequestration and increased agricultural sustainability. We conclude that, although crucial in enabling the identification of plant-derived carbon-utilising microbes, current technologies lack the high-throughput ability to quantitatively apportion carbon use by phylogentic groups and its use efficiency and destination within the microbial metabolome. It is this information that is required to inform rational manipulation of the plant–soil system to favour organisms or physiologies most important for promoting soil carbon storage in agricultural soil.

## THE SOIL CARBON CYCLE AND MICROBIAL DECOMPOSERS: FUNDAMENTAL PRINCIPLES

All living organisms depend on the supply of necessary elements from the Earth. Since the Earth is a closed system with a finite supply of essential elements such as hydrogen (H), oxygen (O), carbon (C), nitrogen (N), sulfur (S) and phosphorus (P), recycling of these elements is fundamental to avoid exhaustion. Microbes are critical in the process of breaking down and transforming dead organic material into forms that can be reused by other organisms. This is why the microbial enzyme systems involved are viewed as key ‘engines’ that drive the Earth's biogeochemical cycles.^1^

**Figure 1:**
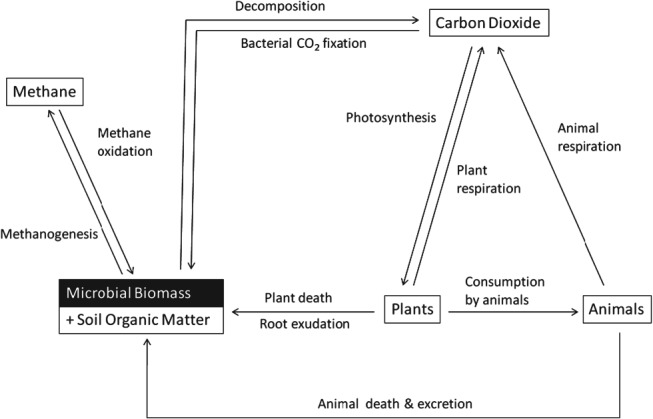
The terrestrial carbon cycle with the major processes mediated by soil microorganisms (adapted from Prosser^125^).

**Figure 2:**
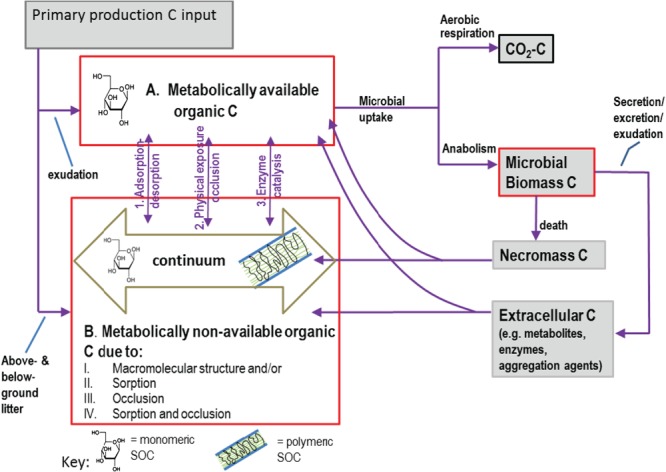
Fate of primary production inputs to soil. Plant-derived organic carbon (after appropriate extracellular depolymerisation) is processed by soil microorganisms to CO_2_, microbial biomass and extracellular substances. Microbial necromass and metabolites are the precursors for stable soil organic matter, while extracellular microbial carbon may also influence the stability of soil organic carbon (SOC). Enzymes may catalyse the depolymerisation of soil macromolecular constituents, while other extracellular substances may promote aggregation and the physical protection of SOC. SOC (red boxes) is depicted as a continuum of structures derived from the progressive decomposition of litter and exudates and includes the microbial biomass carbon. Dissolved and exposed organic carbon (A) is available for microbial cellular uptake and metabolism (catabolism + anabolism) to produce CO_2_ and new biomass respectively. Macromolecular or sorbed or occluded SOC is metabolically non-available (B) but may become available via enzymatic depolymerisation, desorption or exposure (I–III respectively), assuming adequate water, electron acceptors, heat, pH and nutrients for microbial activity.

The terrestrial carbon cycle is dominated by the balance between photosynthesis and respiration.^2^ Carbon is transferred from the atmosphere to soil via ‘carbon-fixing’ autotrophic organisms, mainly photosynthesising plants and also photo- and chemoautotrophic microbes,^3^,^4^ that synthesise atmospheric carbon dioxide (CO_2_) into organic material (Fig. 1). Fixed carbon is then returned to the atmosphere by a variety of different pathways that account for the respiration of both autotrophic and heterotrophic organisms^4^ (Fig. 1). The reverse route includes decomposition of organic material by ‘organic carbon-consuming’ heterotrophic microorganisms that utilise the carbon of either plant, animal or microbial origin as a substrate for metabolism, retaining some carbon in their biomass and releasing the rest as metabolites or as CO_2_ back to the atmosphere (Figs 1 and 2).^5^ Globally, most soils are unsaturated and oxic, so CO_2_ is the main respiration flux. In waterlogged anoxic soils such as rice paddies and peatlands, CO_2_ is reduced by hydrogenotrophic archaea in methanogenesis,^3^,^6^ with the net flux of the methane produced dependent on the relative activity of methanogens (including those fermenting acetate) *versus* the activity of aerobic methane-oxidising bacteria^7^–^9^ (methanotrophs) residing in the surface, oxic layers of soil of such wetland systems and also probably the microbial anaerobic oxidation of methane in anoxic layers.^10^

Soil microbes essentially transfer carbon between environmental compartments to fulfil their fundamental goal: survival through reproduction. Thus, microbes utilise different organic and inorganic forms of carbon as carbon and energy sources. However, the C cycle does not operate independently; it is closely coupled with that of other essential elements for microbial metabolism. This linkage occurs either via the use of the other elements as electron donors and acceptors (e.g. N species ranging from the most reduced, 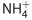, to the most oxidised, 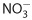) in energy transduction or via their immobilisation and mineralisation as part of multiple essential element-containing biomolecules (e.g. proteins, DNA). Hence the availability of other key elements essential for life, particularly N and P, and other environmental factors such as pH, soil texture and mineralogy, temperature and soil water content control the rate at which microbes consume and respire carbon.^11^ It is these interactions between environmental conditions and biological processes, including primary production, that are chief in controlling the unequal distribution of organic matter across the world's soils.^12^ The largest global concentration of carbon can be found in wet and cool areas in the northern hemisphere, dominated by deep accumulations of peat and permafrost soils,^13^ whereas soils with the lowest carbon content tend to be those of desert biomes,^14^ where low mean annual precipitation limits primary production and encourages the prevalence of aerobic soil conditions.

The objectives of this review are (i) to outline the significance of soil microbial communities to global environmental issues of soil organic matter persistence and climate change through carbon cycle feedbacks, (ii) to briefly describe the main techniques that can be used to apportion below-ground utilisation of plant-derived carbon to specific microbial groups and (iii) to discuss whether application of these techniques can provide the information required to underpin the management of agro-ecosystems for carbon sequestration and increased agricultural sustainability.

## THE SOIL CARBON CYCLE AND MICROBIAL DECOMPOSERS: SIGNIFICANCE AND ENVIRONMENTAL IMPLICATIONS

### Relationship between soil and atmospheric carbon pools

Estimates suggest that global soil organic carbon stocks are equivalent to at least three times the amount of carbon stored in the atmosphere (Table1). About 8% of the total atmospheric carbon pool is exchanged annually between terrestrial ecosystems and the atmosphere via net primary production and terrestrial heterotrophic (predominantly microbial) respiration (Table1). In other words, if soil (microbial) respiration ceased, it would only take about 12 years of primary production at current rates to exhaust atmospheric CO_2_ stocks (if all other components of the carbon cycle are ignored, e.g. oceanic CO_2_ exchange).^15^

**Table 1:** Estimates of the magnitude of soil carbon pools in relation to the atmospheric carbon pool and annual fluxes

	Carbon (Gt or Gt year^−1^)
*Pool*	
Global soil organic carbon (0–300 cm depth)	2344^a^
Northern circumpolar permafrost region soil organic carbon (0–300 cm)	1024^b^
Cropland soil organic carbon (0–300 cm)	248^a^
CO_2_-C in atmosphere	762^c^
*Annual flux*	
Net primary production (photo- and chemosynthesis minus autotrophic respiration)	60^d^
Terrestrial heterotrophic respiration	55^d^
Anthropogenic CO_2_-C (fossil, cement, land-use change)	8^c^

a Jobbágy and Jackson (2000).^14^

b Tarnocai *et al.* (2009)^13^ – a new estimate suggesting significantly more organic carbon in this northern latitude region than reported in previous analysis, e.g. tundra 144 Gt and boreal 150 Gt, by Jobbágy and Jackson (2000).^14^

c Solomon *et al.* (2007)^126^ – estimated for the 1990s.

d Prentice *et al.* (2001)^2^ – estimated for the 1980s.

**Figure 3:**
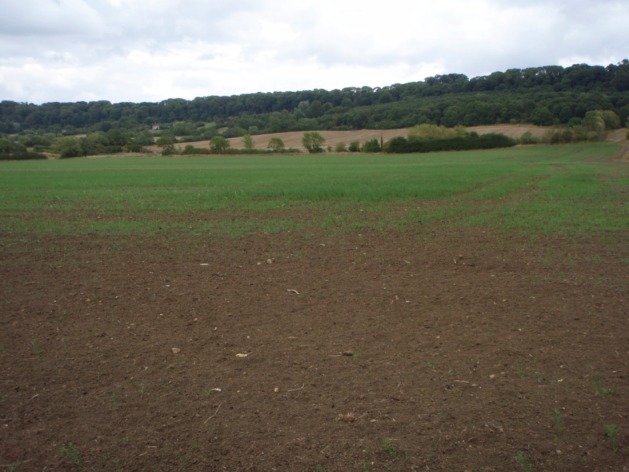
Cereal crop production, Warwickshire, UK (image courtesy of JM Clark). Soils are often seen as simply ‘growing media’ for food crops; however, functioning of microbiological communities within soils not only influences the supply of essential plant nutrients but also has great implications for the global carbon cycle and climate system that determines whether environmental conditions are favourable for crop growth.

At present, terrestrial ecosystems fix, globally, more atmospheric CO_2_ by photosynthesis than they return to the atmosphere through respiration, which includes removing around 25% of global fossil fuel emissions annually.^16^ However, net carbon sequestration varies between locations and is significantly affected by land management. Estimates suggest that 42–78 Gt of carbon have been lost from the world's degraded and agricultural soils owing to human activity in both pre- and post-industrial times (Fig. 3), and land remediation to ‘restore’ some of this lost carbon could make a significant contribution to offsetting fossil fuel emissions.^17^

### Microbial decomposition of plant-derived carbon and persistence of soil organic matter

There are two main routes of input for plant organic carbon to the soil system: (i) above-ground plant litter and its leachates, i.e. dissolved organic carbon washed into the soil from plant material by infiltrating rainfall, and (ii) below-ground root litter and exudation, collectively known as rhizodeposition. The relative magnitude of the various inputs from above and below ground will depend upon plant species and, in soils under agriculture, crop management. Rhizodeposition consists of a continuous flow of carbon-containing compounds from roots to soil. Simple molecules such as sugars, amino acids, sugar alcohols, organic acids and more structurally complex secondary metabolites are among the chemical groups that make up the plethora of root exudates^18^ that can be rapidly (hours to days) respired following their deposition to soil.^19^ By contrast, polymers such as lignin, cellulose and hemicellulose (the typical structural constituents of plant cells^20^) require depolymerisation by extracellular enzymes before they can be taken into the microbial cell and metabolised (Fig. 2).^21^ Of particular note in soil carbon cycling is the role of the mycorrhizal fungi. These range from obligate symbionts that can only obtain carbon from the host plant, i.e. the arbuscular mycorrhizal fungi (AMF), to facultative symbionts that can also mineralise organic carbon, e.g. the ectomycorrhizal fungi (ECM). The AMF symbiosis is found in about 85% of all plant families (typically herbaceous, including many crop species, but also woody),^22^ and experimental evidence suggests that up to 20%^23^–^25^ or even 30%^26^ of total carbon assimilated by plants may be transferred to the fungal partner, with the symbiosis having profound effects on rhizodeposition.^27^ A proportion of the plant carbon that is transferred to the mycelia is very quickly respired back to the atmosphere, and this represents a short-circuit of the soil carbon cycle.^23^,^25^,^26^,^28^,^29^

Soil organic matter (SOM) consists of the continuum from fresh to progressively decomposing plant, microbial and faunal-derived debris and exudates, including the microbial biomass that is responsible for the primary decomposition of the exudate and detrital inputs (Fig. 2).^30^ Traditionally, this continuum has been divided into a series of pools with varying decomposition kinetics, ranging from ‘active’ pools that turn over in months to ‘passive’ pools that turn over in thousands of years. In addition to containing fire-derived ‘black carbon’, the ‘passive’ pool has long been thought to be composed of constituents that get their resistance to decomposition from their humified nature, with the formation of the humified substances resulting from spontaneous condensation reactions between reactive microbial products and biochemically altered structural biomolecules.^31^ However, recent evidence suggests that environmental and biological factors may exert a far greater control on the long-term persistence of SOM than the molecular structure of plant litter inputs and subsequent formation of humus, forcing a re-evaluation of the concept of ‘recalcitrant’ soil humic substances that underpins predictive models of carbon turnover.^12^,^30^ Direct, *in situ* observations have not been able to verify the existence of humic macromolecules in soil, suggesting that the extraction of humic substances from soil may be an artefact of the method used to extract them.^32^ Instead, it is suggested that SOM consists of partially decomposed litter and a significant proportion of microbial necromass (i.e. dead biomass residues)^33^ and that SOM persists or is ‘passive’ owing to its physical disconnection from, or inaccessibility to, the extracellular enzymes, microorganisms and the optimal environmental conditions (e.g. electron acceptors, water, inorganic nutrients) needed for decomposition as a result of entrapment within soil aggregates and/or sorption to soil mineral phases. Therefore, in addition to their role in the breakdown and release of CO_2_ from organic matter, soil microbes contribute to the formation of persistent SOM via their necromass. Additionally, the activities of soil microorganisms may contribute to the stabilisation of soil organic carbon through promotion of the formation of microaggregates, within which SOM may be physically protected from decomposition.^34^,^35^ In particular, glomalin, a glycoprotein produced by the hyphae of AMF, has received much attention with respect to its suggested role in the stabilisation of microaggregates.^36^,^37^ Similarly, other hydrophobic proteins produced by mycorrhizal fungi and filamentous bacteria, such as hydrophobins and chaplins, have been associated with microaggregate formation and stabilisation.^36^,^37^

### Soil carbon cycle, microbial decomposers and climate change

As mentioned, humans have heavily perturbed the carbon cycle during the industrial period through inputs of CO_2_ to the atmosphere, mainly via combustion of fossil fuel and conversion of natural ecosystems to agricultural land (Table1).^2^,^38^ The consequences of human actions for the global climate are still uncertain, partly owing to our limited understanding about soil respiration and its representation in Earth system models.^38^–^40^ Microbial contributions to climate change through carbon cycle feedbacks are far from straightforward, complicated by direct and indirect effects and interactions with other factors^41^ (also reviewed in Bardgett *et al.*^42^ and Singh *et al.*^43^).

An example of a simplified direct positive feedback to global warming is that microbial activity, and therefore organic carbon decomposition and CO_2_ released by respiration, may be accelerated in response to an increase in temperature.^11^,^44^–^50^ Analyses of field observations made across the globe point to a link between increased respiration flux from land and increased temperatures.^51^

An example of an indirect positive feedback to elevated CO_2_ is a consequence of the carbon fertilisation of primary (photosynthetic) production, whereby increased atmospheric CO_2_ stimulates photosynthesis^52^,^53^ and the release of root exudates, which in turn means more labile carbon available for microbial decomposition and respiration.^53^–^57^ Moreover, increased root deposition of easily available exudates may ‘prime’ the turnover of less available SOM constituents that otherwise would not be subject to decomposition.^58^–^63^

Understanding how the balance between terrestrial ecosystem sinks (i.e. photosynthesis) and sources (i.e. respiration, including microbial respiration) of atmospheric CO_2_ will be affected in an elevated CO_2_ world is still one of the main uncertainties in understanding the coupled carbon–climate system.^4^,^39^,^41^,^47^,^57^ The uncertainty increases when soil nitrogen and its availability to plants are taken into consideration, with contradicting indications.^64^ Some modelling studies have suggested that there may be a point during this century where terrestrial ecosystems shift from a net sink to a net source of atmospheric CO_2_,^45^,^54^,^65^ possibly reflecting the scenarios of increased microbial respiration;^45^ however, these models are still in an early stage of development.^55^ Questions still remain about the actual temperature sensitivity of soil (microbial) respiration and how this sensitivity is modified by other environmental factors such as changes in soil moisture during droughts and nutrient limitations and physical protection of organic matter in aggregates or by sorption.^4^,^11^,^30^,^66^,^67^ This problem is exacerbated by the diversity of soil ecosystems across the world, which vary in their function owing to differences in their forming factors: parent material, topography, climate, organisms and time. Particular concerns have been raised about peatlands and permafrost soils, where climatic conditions that cause the accumulation or preservation of organic material may not be favourable under a future climate, resulting in the release of significant quantities of carbon to the atmosphere.^11^,^39^ Further research in this area is an urgent priority if we are to be able to predict impacts and feedbacks between climate change and the global carbon cycle.^39^,^67^

## METHODOLOGIES FOR TRACKING CARBON FLOW BELOW GROUND TO MICROBIAL GROUPS

Despite the important role of soil microbes in the carbon cycle and the environmental implications of carbon cycle–climate change feedbacks, most carbon cycle models treat the soil microbial biomass as a black box.^68^ The majority of models calculate soil respiration using first-order kinetics, where decomposition (and CO_2_ flux) is proportional to the size of the carbon pool defined by an empirically derived decomposition rate constant that captures the net effect of microbial activity under specific conditions.^60^,^69^ Pools typically represent ‘slow’ and ‘passive’ organic matter fractions; and models differ in terms of the number of conceptual pools used (one to nine) and addition of rate modifiers that account for changes in temperature, moisture, etc. (see e.g. Freidlingstein *et al.*^55^). Recent studies have called for modifications to these traditional ‘black box’ SOM models to include more explicit representations of microbial community and functions that control decomposition. However, much of this detail is absent because the basic processes are still poorly understood.^11^

In empirical microbial ecology we are now beginning to be able to unpack the microbial biomass ‘black box’ and to identify which microbial groups are responsible for the turnover of plant-derived inputs to soil. The methods available to do this generally involve the combination of either stable (SIP) or radioactive (RIP) isotope probing and molecular ecology techniques (for a thorough review on methodologies for linking microbial identity to environmental processes, see Gutierrez-Zamora and Manefield^70^). In SIP or RIP the stable or radioactive isotope (^13^C or ^14^C respectively) is used to track the microbial fate of a labelled carbon source (or sources). As a result of anabolic processes, the carbon label becomes incorporated into the biomolecules of those microbes actively decomposing the carbon source of interest. In nucleic acid-SIP the resulting ‘heavier’ 13C DNA or RNA is fractionated by isopycnic density gradient ultracentrifugation from unlabelled nucleic acids and used as a template for downstream analysis. When ^14^C is used as the label, RIP can be detected with microautoradiography (MAR) and combined with fluorescence *in situ* hybridisation (FISH), termed FISH-MAR, to enable allocation of carbon utilisation to specific microbial ribotypes with the use of fluorescent rRNA-targeted oligonucleotide probes. The use of an autoradiographic emulsion causes silver grains to form in areas immediately adjacent to radioactive cells (proof of ^14^C substrate incorporation into the microbial biomass), whereas cell fluorescence after exposure of slides to FISH probes under hybridisation conditions is used for the phylogenetic identification.

Nucleic acid-SIP was first applied over a decade ago, and the majority of studies have focused on non-planted systems. Methanol-utilising microorganisms in soil were studied first with DNA-SIP,^71^ and subsequent studies have built on this work and used DNA-SIP to link function and identity with respect to the cycling of methane^6^,^7^,^72^,^73^ and the biodegradation of organic pollutants.^74^–^77^

Phenol-utilising microorganisms in wastewater treatment plants were first studied with rRNA-SIP.^78^ Since then, RNA-SIP has successfully been used in numerous studies to track the carbon utilisation in specific microbial groups in diverse ecosystems (e.g. rivers, tidal flats, aquifers, groundwater) with respect to carbon biogeochemical processes such as methanotrophy,^9^,^72^,^79^ degradation of xenobiotics^80^–^84^ and other ecosystem functions.^79^,^84^,^85^

The application of nucleic acid-SIP to trace, *in situ*, the microbial fate of rhizodeposit carbon in soil involves the growth of plants in a ^13^C-CO_2_ atmosphere to promote ^13^C labelling of photosynthate and therefore rhizodeposition. For successful nucleic acid-SIP, a high proportion of ^13^C-labelled substrate incorporation is required in order to achieve sufficient separation of ‘heavy’ and ‘light’ nucleic acids. With the exception of the use of DNA-SIP to study endophytes that are, by definition, in intimate association with plant root systems,^86^ this level of labelling is sometimes difficult to achieve when tracking the microbial fate of plant-derived carbon in the rhizosphere because of dilution with plant ^12^C and the native ^12^C SOM.^87^ This sensitivity problem was demonstrated in a study^88^ where, although the labelling of bacterial nucleic acids with ^13^C following ^13^CO_2_ incubation of grassland turfs occurred, the amount of labelling was indeed too low, preventing the separation of ^13^C- from ^12^C-nucleic acids (for reviews on methodological considerations, see Manefield *et al.*^87^ and Neufeld *et al.*^89^). As rRNA is turned over independently of cell replication with a high copy number within the microbial cell, rRNA-SIP has a greater sensitivity than DNA-SIP (reviewed in Whiteley *et al.*^90^) and has therefore been used more successfully to directly track plant-derived carbon to microorganisms in the rhizosphere.^3^,^26^,^29^,^91^–^93^ The promise of mRNA-SIP has also been recently explored for understanding the links between root exudation and bacterial gene expression in the rhizosphere.^94^ The SIP approach has been complemented and extended to track plant-derived carbon into biochemical markers other than nucleic acids, such as proteins (protein-SIP;^95^ for reviews, see Seifert *et al.*^96^ and von Bergen *et al.*^97^) and phospolipid fatty acids (PLFA-SIP).^98^–^100^ Drigo *et al.*,^26^ Hannula *et al.*^92^ and Dias *et al.* (2013)^101^ have recently combined RNA-SIP, neutral lipid fatty acid (NLFA)-SIP with neutral (NLFA) and phospholipid (PLFA) lipid fatty acids biomarker analyses and/or PLFA-SIP with real-time polymerase chain reaction (PCR) and community fingerprinting techniques to examine how elevated CO_2_ or plant genetic modification alters the destination of photosynthetically fixed carbon with respect to its utilisation by AMF and mycorrhizosphere bacterial and fungal species.

FISH-MAR was first demonstrated in 1999 by two separate research groups that managed to visualise the incorporation of 14C-labelled substrates in probe-detected bacteria under the microscope.^102^,^103^ Since then, it has been used mainly to study *in situ* physiology of bacteria in biofilms^104^ and activated sewage sludge with enhanced biological phosphorus removal.^105^–^107^ Although FISH-MAR has greater sensitivity than DNA- or RNA-SIP, as detection of substrate incorporation is not restricted to analysis of a specific biomolecule (in contrast to nucleic acid-SIP), it is limited, firstly because the microbial groups to be targeted need to be known (and therefore selected with the use of appropriate molecular probes) in advance and secondly owing to the fact that its application is restricted to either single or small clusters of cells (reviewed in Wagner *et al.*^108^). Limitations on the number of different fluorophores that can be detected simultaneously also restrict the number of microbial groups that can be targeted at the same time in FISH-MAR.^109^ However, the development of radioactively labelled RNA-targeted isotope arrays to study multiple microbial populations for their ability to consume a radioactive substrate in activated sludge samples has given promising results.^110^ The isotope array concept has recently been expanded in isotope rRNA-targeted oligonucleotide microarrays (PhyloChips), containing a much larger number of probes, to reveal substrate consumption profiles of *Rhodocyclales* spp. in activated sludge.^111^ Isotope arrays have, to our knowledge, not yet been used in soil-based studies.

Returning to *in situ* hybridisation-based techniques, FISH-MAR has been used in combination with catalysed reporter deposition^112^ to improve signal detection in oligotrophic prokaryotes, and quantitative (Q)-FISH-MAR^104^ has been developed to quantify cell-specific carbon uptake in probe-targeted bacterial groups. In addition, the combination of FISH with other methodologies has given birth to further hyphenated techniques such as FISH-SIMS^113^,^114^ (the combination of FISH with secondary ion mass spectrometry) and FISH-RAMAN^115^ (the combination of FISH with Raman microspectroscopy). Both SIMS and Raman microspectroscopy can be applied to characterise cellular incorporation of ^13^C-labelled substrates, negating the requirement for experiments using ^14^C-CO_2_ pulse chase and attendant safety concerns with respect to use of radioactivity. In FISH-SIMS the FISH-probed identification of microbial cells is coupled to SIMS, which determines the isotopic composition/incorporation of the targeted cells after a caesium ion beam is directed on their surface. In FISH-RAMAN the FISH identification is coupled to highly resolved Raman confocal spectra, and cells that are ^13^C-labelled through anabolic incorporation of the isotope exhibit key ‘red-shifted’ spectral peaks highly correlated with their ^13^C content. The FISH-RAMAN method was initially used in naphthalene-degrading groundwater samples^115^ and can be quantitative with appropriate calibration. The same authors expanded their research by combining RNA-SIP and FISH-RAMAN to fully explore naphthalene degradation of the polluted groundwater.^83^ Although not yet applied to the root zone, to our knowledge, both FISH-SIMS and FISH-RAMAN have considerable promise for use in rhizosphere carbon flow tracking experiments.

## POTENTIAL FOR MANIPULATING CARBON DYNAMICS IN AGRICULTURAL SYSTEMS

The intensive cultivation of soils under agriculture results in the loss of soil carbon due to (i) the acceleration of decomposition through improved aeration and the exposure of physically protected organic matter as a result of tillage and drainage and (ii) the reduction of primary production inputs to soil through the removal of plant biomass during harvest. As already mentioned, it is estimated that somewhere in the range of 42–78 Gt of carbon^17^ that was historically stored in the soil system has been lost as a result of the intensive cultivation of soils, and the capacity for agricultural soils to regain this lost carbon is currently being discussed as one potential contribution to atmospheric carbon remediation and mitigation of climate change.^17^,^116^

The size of the C store in soil depends on the interactions between (i) the quantity and quality of primary production inputs and (ii) the fate of these inputs once they have entered the soil in the short and long term. Strategies with respect to the management of soil for C sequestration therefore involve increasing the quantity of primary production, and indeed other organic inputs into the system; this possibility has been widely debated with respect to breeding crop plants with more extensive root systems^117^ or altered physiological traits, cover- and inter-cropping, increasing the return of crop residues to soil^118^ and addition of amendments such as compost or biochar.^119^–^121^ However, the addition of crop residues and other amendments, while increasing soil organic C, does not usually transfer C additional to that already fixed from the atmosphere to land (depending on the alternative fate of the amendment) and therefore the end point of such practice does not necessarily qualify as ‘soil C sequestration’ under the strictest definitions of this term.^118^ A second strategy involves manipulating the fate of the inputs once added to the soil. On entering soil, inputs may (after extracellular depolymerisation in the case of macromolecular constituents) be taken up by the soil microbial biomass and the C partitioned for use in the production of biomass (subsequently necromass), excretions and secretions (e.g. extracellular enzymes) and respiration (Fig. 2). The aim of this second strategy is to encourage the processing of plant-derived C to biomass and metabolite precursors of soil organic matter or to secretions that promote the physical protection of C substrates against decomposition rather than to CO_2_; in other words, to increase the C use efficiency of the microbial biomass potentially by manipulation of the quality of rhizodeposit inputs or edaphic environmental conditions that have a moderating effect on soil microbial physiology.

We know that climatic and abiotic soil factors (e.g. clay content) influence soil C cycling; however, the identity of soil microorganisms, as the primary decomposers of plant-derived C, is likely to significantly influence the fate of C inputs to soil. The extent to which climatic effects on soil C cycling are confounded with microbial adaptation to certain environmental niches is currently unknown. There is evidence (reviewed in Six *et al.*^122^) to suggest that the relative abundance of fungi and bacteria may be important, with more stable carbon being formed in soils with high fungal/bacterial biomass ratios. That fungi have a higher C use efficiency than bacteria and therefore form more biomass per unit of C utilised and also a biomass (subsequently necromass) of a more recalcitrant nature are the suggested mechanisms for the greater accumulation of fungal SOM, although both these mechanisms require further study.^122^ There have also been some studies (reviewed in Nielsen *et al.*^123^) that have reported relationships (positive and negative) between soil biodiversity and C cycling processes such as respiration, but these have generally focused on total species richness as the biodiversity measure and not the richness or identity of those species processing the carbon *in situ*.

At this stage it is not clear how the diversity and identity of those microorganisms using plant C influence the fate of that C. In addition, the edaphic abiotic factors controlling microbial C utilisation efficiency have not been thoroughly characterised.^124^ Ultimately it is not clear to what extent rhizosphere microbes within agricultural systems can be manipulated for C sequestration. If the community structure of plant C-utilising microbes is important for C fate, then the next step is to understand which are the most important groups that control soil storage with respect to expression of specific functions (e.g. metabolite production) and the proportion of the plant C inputs they are responsible for processing. This information is essential for both conceptualisation and parametrisation of the next generation of SOM models. To do this, we need to be able to *quantitatively* apportion plant C to specific microbial groups *in situ* and to partition its use (i.e. for biomass/metabolite/CO_2_ production) within that group.

We conclude that the methodologies outlined in this paper, although crucial in enabling the identification of plant-derived carbon-utilising microbes, lack the high-throughput ability to do this because of their reliance on extracted biomolecules (nucleic acid-SIP, protein-SIP, PLFA-SIP), precluding the ability to study the partitioning of carbon at the whole cell level, or because they are limited to the study of a small number of cells (FISH-SIMS, FISH-RAMAN). The challenge is the development of new methodologies that allow quantification of microbial use efficiency and destination of plant carbon (within phylogenetic groups and the metabolome) to enable a step-change level of understanding. The ultimate benefits from this investment will be the knowledge to inform manipulation of the plant–soil system to favour organisms or physiologies most important for promoting soil carbon storage across the diverse conditions present in the global agricultural land.
